# 热脱附-气相色谱-质谱法测定石化行业废气中环氧乙烷和乙醛

**DOI:** 10.3724/SP.J.1123.2025.01016

**Published:** 2025-12-08

**Authors:** Yingjie LI

**Affiliations:** 生态环境部城市大气复合污染成因与防治重点实验室，上海市环境科学研究院，上海 200233; Key Laboratory of Formation and Prevention of Urban Air Pollution Complex，Ministry of Ecology and Environment，Shanghai Academy of Environmental Sciences，Shanghai 200233，China

**Keywords:** 热脱附-气相色谱-质谱, 挥发性有机化合物, 环氧乙烷, 乙醛, thermal desorption-gas chromatography-mass spectrometry （TD-GC-MS）, volatile organic compounds （VOCs）, ethylene oxide, acetaldehyde

## Abstract

环氧乙烷和乙醛是石化行业排放废气中挥发性有机化合物（VOCs）的重要组分，乙醛对臭氧生成的贡献潜力高于环氧乙烷几个数量级，需对其精准识别和定量。因为环氧乙烷和乙醛是同分异构体且挥发性相近，常规分析方法难以实现两者的有效分离和准确定量。本研究基于热脱附-气相色谱-质谱技术，通过优化气相色谱柱升温程序、热脱附温度和热脱附流速，建立了能够同时收集、高效分离并准确定量环氧乙烷和乙醛的分析方法。在优化的分析条件下，环氧乙烷和乙醛在1~10 ng/管范围内呈现良好的线性关系，相关系数均在0.99以上，方法检出限分别为0.16 ng/管和0.021 ng/管，目标物的热脱附效率大于95%；目标物在2、5和10 ng/管加标水平下的回收率为80.3%~106.8%，相对标准偏差≤9.2%。采用所建立方法对某石化企业生产装置末端排放口废气进行检测，发现废气中同时存在环氧乙烷和乙醛，且两者浓度不同。该方法不仅为污染源环氧乙烷和乙醛监测提供了一种可靠的技术手段，也可用于其他排放源或环境空气中环氧乙烷和乙醛的监测，为活性VOCs 精准管控提供基础数据支持。

## Abstract (en)

Ethylene oxide （EO） and acetaldehyde are key components of volatile organic compounds （VOCs） emissions in the petrochemical industry. The ozone formation potential of acetaldehyde is several orders of magnitude higher than that of EO. Therefore， accurately identifying and measuring these compounds is essential for developing effective ozone control strategies. However， separating EO from acetaldehyde presents a considerable analytical challenge due to their isomeric nature and comparable volatilities. In this study， a novel analytical method based on thermal desorption-gas chromatography-mass spectrometry （TD-GC-MS） technology was developed to simultaneously collect， separate， and quantify EO and acetaldehyde. Optimization was performed on the GC column temperature program， thermal desorption temperature， and thermal desorption flow rate to achieve effective separation. Compounds were separated on a TG-624 SiIMS GC column （60 m×0.25 mm×1.4 μm） with a carrier gas flow rate of 1.2 mL/min. To separate EO from acetaldehyde， the GC column temperature was specifically optimized to enhance their differences in desorption rates from the stationary phase. The temperature was initially held at 30 ℃ for 3 min， then ramped at 5 ℃/min to 120 ℃， held for 3 min. Both full scan and selected ion monitoring modes were employed for target detection. During thermal desorption， the thermal desorption temperature was set at 180 ℃， the cold trap temperature was set at -30 ℃ for capturing desorbed targets， and the secondary desorption flow rate was set at 16 mL/min with the corresponding split ratio of 12.3. The method’s performance was evaluated under optimized experimental conditions. Within the range from 1 to 10 ng/tube， the method showed strong linearity with correlation coefficients above 0.99 for EO and acetaldehyde. Method detection limits were determined to be 0.16 ng/tube for EO and 0.21 ng/tube for acetaldehyde. Thermal desorption efficiencies for both targets exceeded 95% with samples spiked at 2 and 10 ng/tube. Recovery efficiencies spiked at 2， 5 and 10 ng/tube ranged from 80.3% to 106.8%， with relative standard deviations ranging from 4.5% to 9.2%. This method was applied to VOCs samples collected from aftertreatment exhaust streams of two petrochemical units， where different concentrations of EO and acetaldehyde were detected. This study thus established a reliable method for the simultaneous collection， identification and quantification of EO and acetaldehyde in petrochemical emission matrices. Furthermore， this method can also be used for monitoring these compounds in diverse emission sources and ambient air， thereby providing essential data to support the management and control of reactive VOCs.

臭氧（O_3_）污染是当前我国大气污染防治面临的最突出问题。环境空气中的O_3_是挥发性有机化合物（VOCs）和氮氧化物（NO *_x_* ）在光化学反应作用下生成的二次污染物。众多研究表明，VOCs和NO *_x_* 减排是控制O_3_污染的有效途径^［1-3］^。石化化工行业不仅是VOCs的主要排放源，其排放的VOCs组分十分复杂。不同VOCs组分排放进入环境空气后，因其光化学反应活性不同，对O_3_生成的贡献可能存在数量级差异^［4］^。因此，精准识别和定量活性VOCs组分是实现科学和精准治污的基础。

环氧乙烷和乙醛是石化行业重要的基础化工原料，是乙醇胺、合成橡胶、增塑剂等有机化学品制造过程中释放的主要VOCs组分^［5］^。乙醛具有较强的化学反应活性^［6］^，不仅是需要重点管控的O_3_前体物，还因其具有强烈刺激性气味，也是石化化工园区重点管控的异味物质之一^［7］^。环氧乙烷的化学反应活性相对于乙醛较弱，如乙醛的最大增量反应活性（MIR）^［5］^为6.34 g O_3_/g VOC，而环氧乙烷仅为0.037 g O_3_/g VOC^［4］^。该值意味着当大气中的环氧乙烷和乙醛浓度相同时，乙醛生成O_3_的能力（VOCs组分浓度与MIR的乘积）是环氧乙烷的170倍。研究表明，基于大气反应活性的VOCs组分管控是实现O_3_污染控制的有效路径^［4，8］^。因此，准确定量污染源中环氧乙烷和乙醛的排放情况，可为活性VOCs组分精细化管控策略制定提供科学依据。

气相色谱-质谱法（GC-MS）是定量VOCs常用的分析方法，通常采用苏玛罐或热脱附管收集含有环氧乙烷和乙醛的VOCs样品，直接进样至GC-MS系统进行分析。环氧乙烷和乙醛的沸点分别为20 ℃ 和11 ℃，挥发性差别不大，色谱峰极易重叠，且二者为同分异构体（图1），其质谱图离子碎片特征相同（https://webbook.nist.gov）。因此，常规GC-MS方法难以对重叠色谱峰中的这两个化合物进行识别和分别定量^［9-11］^，通常仅能测定环氧乙烷和乙醛的总量。质子转移反应质谱法（PTR-MS）也是测定乙醛的常用方法之一^［12-15］^，PTR-MS没有气相色谱分离单元，仅通过相对分子质量进行识别，该方法不能区分具有相同相对分子质量的环氧乙烷和乙醛，其测定结果仍为两者的总量。衍生法^［16-20］^采用含有二硝基苯肼吸附剂的吸附柱收集乙醛，乙醛与吸附剂反应生成其他稳定的衍生物，再采用液相色谱与质谱联用进行分析，可以实现乙醛的单独测定，环氧乙烷含量则通过常规GC-MS或PTR-MS方法测定的两者总量与乙醛含量的差值得到。但是，衍生法收集的目标物需使用溶剂进行萃取，烦琐的萃取过程不仅耗时耗力，也易引入污染和误差。

**图1:**
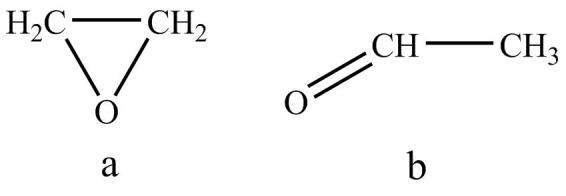
（a）环氧乙烷和（b）乙醛的分子结构

当前，乙醛已被列为石化化工行业排放VOCs的常规监测组分之一，而对环氧乙烷的排放情况鲜有报道^［21］^。事实上，石化化工园区存在环氧乙烷排放源，如部分化工装置周边环境空气中环氧乙烷浓度可达几至几百mg/m^3［22，23］^，远远超过环氧乙烷的职业卫生标准限值（<2 mg/m^3^）。如采用常规GC-MS方法分析，因不能对环氧乙烷进行有效识别，可能导致测定的乙醛浓度偏高。这种高估会影响对O_3_生成具有重要贡献的活性VOCs组分的准确评估，从而影响制定精准的管控措施。

热脱附（TD）是将VOCs样品收集到吸附剂上，在一定的气流下加热吸附剂萃取目标物的过程。由于其样品收集和预处理过程简单，且设备轻巧，TD联合GC-MS（TD-GC-MS）已成为检测环境空气及固定污染源废气中VOCs常用的分析方法^［24-27］^。本研究以TD-GC-MS为主要分析手段，通过优化色谱柱升温程序、TD热脱附温度、热脱附流速等参数，实现环氧乙烷和乙醛的高效分离和定量分析，并将建立的方法用于测定某石化企业特定装置排放废气中环氧乙烷和乙醛的排放情况。

## 1 实验部分

### 1.1 仪器、试剂与材料

8890/5977B气相色谱-质谱联用仪购自美国Agilent公司；TD-100Xr热脱附仪、不锈钢TD管和冷阱管、TC-20^TM^不锈钢热脱附管老化仪购自英国Markes公司；不锈钢苏玛罐、苏玛罐清洁系统（3100A）和限流器（CS1200）购自美国Entech公司；质量流量计（D08-1F0）和压力控制阀（CS 200）购自北京七星华创流量计有限公司。

环氧乙烷溶液（10 000 mg/L溶于二甲基亚砜，99.5%）购自上海安谱实验科技有限公司；乙醛溶液（1 000 μg/L溶于乙腈，99.5%）购自美国侨怡生物科技有限公司；乙腈溶液（纯度≥99.5%）、石墨化炭分子筛吸附剂、TD管内金属网和玻璃纤维购自Sigma-Aldrich公司（上海）。高纯氮气（99.999%）和高纯氦气（99.999%）购自林德工业气体有限公司（上海）。

### 1.2 实验方法

#### 1.2.1 标准溶液的配制

分别取一定量的环氧乙烷和乙醛标准溶液于乙腈中，得到质量浓度为5 ng/μL的环氧乙烷和乙醛标准溶液；取一定量的环氧乙烷和乙醛标准溶液混合于乙腈中，再经乙腈逐级稀释，最终得到质量浓度分别为1、2、5和10 ng/μL的系列混合标准溶液。

#### 1.2.2 TD管的准备与保存

TD管内吸附剂在实验室内装填完成，吸附剂由在管两端装填的金属网（TD管）或玻璃纤维（冷阱管）固定^［28］^。TD管内装填的石墨化炭分子筛的质量为420 mg；冷阱管内装填的石墨化炭分子筛的长度为420 mm。新装填完成的TD管需在老化仪中，以200 ℃和50 mL/min的高纯氦气吹扫5 h；后续使用，在200 ℃下吹扫1 h。老化后的TD管用锡箔纸包裹，于4 ℃冰箱保存，3天内进行样品采集。

#### 1.2.3 TD管实验室样品准备

TD管中加入1.0 μL系列浓度的环氧乙烷和乙醛标准溶液，以50 mL/min的流速用高纯氮吹扫5 min，以除去溶剂溶液。

#### 1.2.4 样品采集

采用不锈钢苏玛罐分别收集某石化企业生产乙醇胺（EOA）和乙氧基化（EOD）装置末端排放的VOCs样品。EOA装置通过乙烯与氧气反应生成环氧乙烷，再进一步与水反应生成乙二醇；EOD装置以环氧乙烷为原料生产环氧乙烷衍生物。采样参考了美国环保署发布的方法^［29］^，采样前使用专用的苏玛罐清洁系统清洗苏玛罐。使用限流器设置苏玛罐采样时间为1 h。通过一个质量流量计、压力控制阀和聚四氟乙烯管连接TD管和苏玛罐。设定TD管采样流速50 mL/min，TD管样品从苏玛罐采集，采样时间为20 min。

### 1.3 分析条件

#### 1.3.1 热脱附条件

在热脱附前，使用高纯氦气以60 mL/min的流速在室温下吹扫TD样品管1 min，以去除样品中的水分和氧气。随后，以高纯氦为载气，在180 ℃下以60 mL/min的流速进行首次热脱附（3 min），释放的环氧乙烷和乙醛被冷阱管在‒30 ℃下富集。热脱附完成后，冷阱管快速升至180 ℃，在16 mL/min的流速下进行二次热脱附（3 min）。二次热脱附的环氧乙烷和乙醛经由传输管线传输至气相色谱柱前端，传输线温度设置为200 ℃。

#### 1.3.2 气相色谱-质谱条件

色谱柱：TG-624 SiIMS毛细管柱（60 m×0.25 mm×1.4 μm，美国ThermoFisher公司）；载气：高纯氦；流速：1.2 mL/min；升温程序：初始温度30 ℃，保持3 min，然后以3 ℃/min升温至120 ℃，保持3 min。

离子源：电子轰击源（EI）；电离电压：70 eV；离子源温度：250 ℃；传输线温度：200 ℃。扫描模式：全扫描模式（*m/z* 20~100）识别环氧乙烷和乙醛；选择性离子监测（SIM）模式进行分析方法的性能评价和目标物定量；环氧乙烷和乙醛的定量离子为*m/z* 29。

## 2 结果与讨论

### 2.1 色谱柱升温程序优化

气相色谱柱是气相色谱的核心部件，主要利用色谱柱内固定相对目标物吸附和解析能力的不同，实现目标物的分离。因此，可以通过优化气相色谱柱升温程序，影响目标物在色谱柱的解析速度，从而优化目标物的分离效果^［30］^。

本研究首先采用罐采样/GC-MS方法（HJ 759-2015），并结合文献［^9^］的色谱条件，对苏玛罐采集的VOCs样品进行分析。选用DB-624色谱柱（60 m×0.25 mm×1.4 μm）；流速为1.2 mL/min；色谱柱升温程序的初始温度为31 ℃，保持7 min，然后以5 ℃/min升至120 ℃，保持3 min，再以6 ℃/min升至200 ℃，保持9 min^［9］^。结果如图2所示，仅在16.48 min处观察到一个相对较宽的色谱峰，其质谱图与标准质谱库乙醛和环氧乙烷的谱图（https://webbook.nist.gov）特征相同。因此，此处色谱峰应为环氧乙烷和乙醛的重叠峰，表明该方法未能将环氧乙烷和乙醛有效分离。

**图2:**
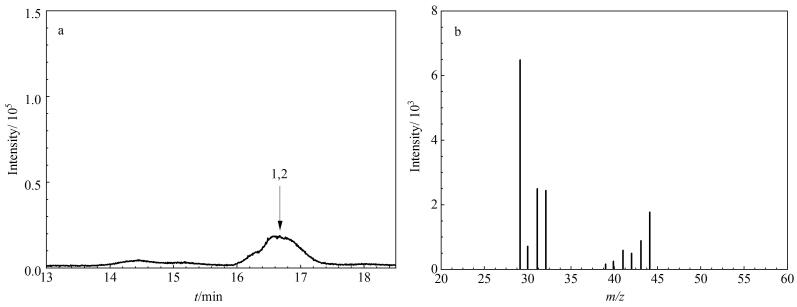
采用罐采样/GC-MS方法^［9］^时环氧乙烷和乙醛的（a）总离子流色谱图和（b）质谱图

在此基础上，本研究将GC色谱柱初始温度降至30 ℃保持3 min，然后以3 ℃/min升至120 ℃保持3 min。为准确识别这两个目标物在色谱柱中分离的先后顺序和相应色谱峰保留时间，在全扫描模式下，依次单独分析了乙醛、环氧乙烷和两者的混合标准溶液。单独分析乙醛时，7.73 min处观察到的色谱峰质谱图与标准质谱库乙醛质谱特征相同，表明此处的色谱峰为乙醛（图3a）；同理，确定了环氧乙烷的色谱峰保留时间为8.47 min（图3b）；分析环氧乙烷和乙醛的混合标准溶液时，在7.73 min和8.47 min处出现的色谱峰（图3c），其质谱特征与标准质谱图库环氧乙烷和乙醛的质谱相同。因此推断7.73 min和8.47 min处的色谱峰分别为环氧乙烷和乙醛，表明该升温程序能有效分离这两种化合物。与常规GC-MS方法比，本研究采用较低的初始温度和升温速率，强化两者在色谱柱解析速度上的差异，是实现分离的关键。

**图3:**
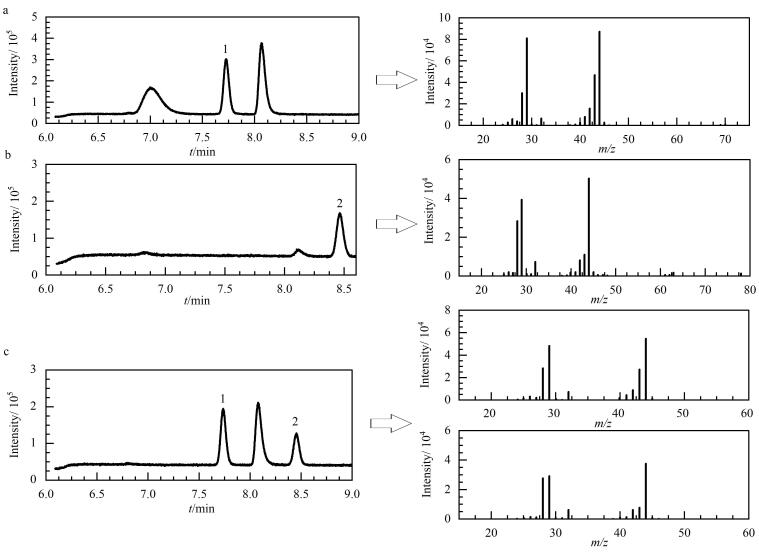
采用TD-GC-MS方法时（a）环氧乙烷、（b）乙醛及其（c）混合标准溶液的总离子流色谱图和质谱图

### 2.2 TD运行参数优化

Li等^［28，31］^的研究表明，TD热脱附温度和冷阱管热脱附（二次热脱附）载气流速是影响目标化合物脱附效果的主要因素。本研究采用1.2.1节配制的混合标准溶液，对以上参数进行了优化，并以环氧乙烷和乙醛色谱峰响应面积最大为基准确定最优值。

温度是影响目标物热脱附效果的重要参数^［28］^。热脱附温度越高，目标物的热脱附效率越高，但过高的脱附温度会导致目标物分解，从而降低目标物的响应强度^［28］^；而热脱附温度偏低又会造成目标化合物解析不完全，影响分析结果的准确性^［28，32］^。本研究分别设置热脱附温度为160、170、180、190和200 ℃，分析了不同温度对环氧乙烷和乙醛色谱峰面积响应的影响。同时，设置一次热脱附的载气流速为60 mL/min，热脱附时间为3 min，二次热脱附流速为20 mL/min，二次热脱附时间和温度与一次热脱附相同。结果如图4a所示，环氧乙烷和乙醛的色谱峰响应面积在180 ℃ 时出现峰值。因此，后续分析中设置热脱附温度为180 ℃。

**图4:**
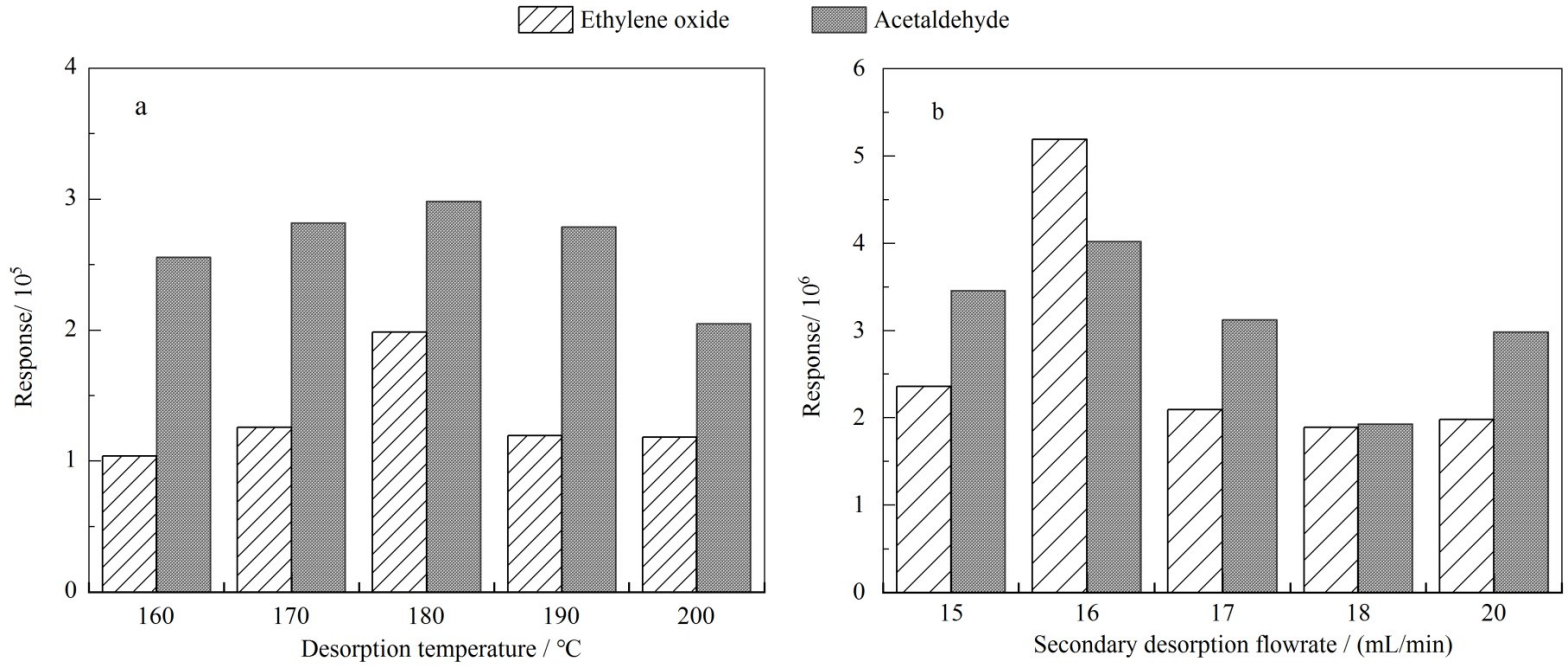
不同（a）热脱附温度和（b）二次热脱附流速下环氧乙烷和乙醛的色谱峰响应面积

已有研究表明，热脱附流速越大，目标物的回收效率越高^［32］^。然而，在气相色谱柱流速一定的情况下，二次热脱附流速越大，分流比将相应增大，进入质谱检测器目标物则相应减少，最终导致目标物检出限降低；但二次热脱附流速较低，会导致目标物在冷阱管残留，降低回收效率^［32］^。本研究分别设置二次热脱附流速为15、16、17、18和20 mL/min（对应分流比为11.5、12.3、13.2、14.0和15.7），分析了不同脱附流速对环氧乙烷和乙醛色谱峰响应面积的影响。结果如图4b所示，随着脱附流速的减少，环氧乙烷和乙醛色谱峰响应面积先呈增加态势，流速为16 mL/min时出现峰值，随后降低。因此，在后续的分析中，设置二次热脱附流速为16 mL/min。

### 2.3 方法验证

#### 2.3.1 线性范围和检出限

对环氧乙烷和乙醛系列混合标准溶液进行测定，分别以目标物的添加量为纵坐标、峰面积为横坐标绘制校正曲线。如表1所示，环氧乙烷和乙醛在添加量为1~10 ng/管范围内具有较好的线性关系，相关系数（*r* ^2^）分别为0.997 7和0.991 6。

**表1:** 环氧乙烷和乙醛的相关系数、方法检出限、热脱附效率、回收率和精确度

Compound	*r* ^2^	MDL/ （ng/tube）	DEs/%		REs/%			RSDs/%		
			2 ng/tube	10 ng/tube	2 ng/tube	5 ng/tube	10 ng/tube	2 ng/tube	5 ng/tube	10 ng/tube
Ethylene oxide	0.9977	0.16	85	95	82.0‒5.6	80.4‒94.2	86.3‒97.4	9.2	4.2	4.9
Acetaldehyde	0.9916	0.21	100	96	87.4‒106.9	96.9‒103.0	81.2‒92.6	4.7	4.8	4.6

向老化后的TD管内添加1 μL 1.2.1节配制的1 ng/μL混合标准溶液，使目标物的添加量为1 ng/管，进行TD-GC-MS分析。重复7次上述实验计算其标准偏差，方法检出限为标准偏差与3.14的乘积^［28］^，环氧乙烷和乙醛的方法检出限分别为0.16 ng/管和0.021 ng/管。

#### 2.3.2 热脱附效率

向老化后的TD管内分别添加2 ng/μL和10 ng/μL的混合标准溶液1 μL，得到目标化合物添加量分别为2 ng/管和10 ng/管，由TD-GC-MS对加标的样品管进行连续分析，直至检测不到目标物。在本研究中，目标物在TD管第3次运行时均未检出。目标物热脱附效率为第1次运行分析目标物的色谱峰响应面积与两次分析目标物面积加和的比值^［28］^。结果显示，环氧乙烷和乙醛的热脱附效率较好，可达95.3%和95.7%。

#### 2.3.3 加标回收率和精确度

向老化后的TD管内分别添加2、5和10 ng/μL的混合标准溶液1 μL，得到目标化合物添加量分别为2、5和10 ng/管，进行TD-GC-MS分析，重复5次。结果表明，目标物的加标回收率为80.3%~106.8%；精确度由5次平行分析结果的相对标准偏差（RSD）进行评价^［32］^。结果如表1所示，除环氧乙烷在2 ng/管水平下的RSD为9.2%外，其他质量水平的RSD为4.2%~4.9%，表明具有较好的精确度。

### 2.4 实际样品分析

为了验证本方法的适用性，分别使用本研究建立的分析方法和罐采样-GC-MS方法对收集的EOA和EOD装置排放口中环氧乙烷和乙醛进行同步测试。为消除因污染源排放波动引起的误差，TD管内样品采自苏玛罐。本研究方法测定EOA装置排放口环氧乙烷和乙醛的含量分别为0.21 mg/m^3^和1.76 mg/m^3^，EOD装置排放口环氧乙烷和乙醛的含量分别为11.95 mg/m^3^和0.65 mg/m^3^。值得注意是，尽管两装置均以环氧乙烷为原料，但仅在EOD排放口检测到高浓度环氧乙烷。这一差异可能是采用不同末端处理技术所致。EOA装置采用氨高压吸收与水洗洗涤组合的废气处理技术，而EOD采用三级水洗塔技术处理尾气。罐采样-GC-MS法测定的环氧乙烷和乙醛的总含量分别为1.90 mg/m^3^（EOA）和13.47 mg/m^3^（EOD），与本方法测定的两个装置排放口环氧乙烷和乙醛含量的加和（1.97和12.6 mg/m^3^）接近，进一步验证了本方法的有效性。根据我国《石油化学工业污染物排放标准》（GB 31571-2015），废气中环氧乙烷和乙醛的排放限值分别为0.5 mg/m^3^和50 mg/m^3^。本研究中测定的EOD装置排放口环氧乙烷浓度超出了排放限值，然而，若采用传统VOCs分析方法，环氧乙烷和乙醛的总含量（按乙醛计算）仍低于国家排放限值。由此可见，传统VOCs分析方法不仅高估了EOD装置乙醛的排放量，可能削弱部分O_3_管控政策的时效性，还干扰了对环氧乙烷超标排放的有效监管。

## 3 结论

本文基于TD-GC-MS技术建立了一种同时收集和测定环氧乙烷和乙醛的方法。通过优化色谱柱升温程序、TD热脱附温度和二次热脱附流速（即分流比），实现了对环氧乙烷和乙醛的分离与准确定量。该方法具有回收率高、灵敏度高、重复性好等优点。将建立的方法用于某石化行业EOA和EOD装置排放口中环氧乙烷和乙醛的实测，监测到EOD排放口环氧乙烷含量（11.95 mg/m^3^）是乙醛的18倍，表明废气中环氧乙烷和乙醛准确定量十分必要。本研究可为污染源排放环氧乙烷和乙醛准确测定提供方法手段。
